# Verbal abuse and mobbing in pre-hospital care services in Chile

**DOI:** 10.1590/1518-8345.2073.2956

**Published:** 2018-01-08

**Authors:** Varinia Rodríguez Campo, Tatiana Paravic Klijn

**Affiliations:** 1 PhD, Assistant Professor, Facultad de Enfermería, Universidad de Concepción, Concepción, Chile.; 2 PhD, Full Professor, Facultad de Enfermería, Universidad de Concepción, Concepción, Chile.

**Keywords:** Violence, Workplace Violence, Occupational Risks, Health Institutions, Nursing, Pre-Hospital Care

## Abstract

**Objective:**

to determine the perception of verbal abuse and mobbing and the associated factors of paramedic technicians (nursing assistants) and professionals (nurses, midwives, kinesiologists) in the pre-hospital care areas of three regions in the south of Chile.

**Methods:**

descriptive and correlational study was performed within the professional community and a two-stage sample of the paramedic technician population in three regions. The questionnaire “workplace violence in the health sector” (spanish version) was applied after signing the informed consent.

**Results:**

51.4% of professionals and 46.6% of paramedic technicians consider they have been verbally abused during last year. 17.6% of paramedic technicians and 13.5% of professionals perceived mobbing. A low percentage of these events are reported. In only one case of mobbing, the aggressor was legally penalized. No significant differences were found between the job categories and the studied regions.

**Conclusions:**

A high percentage of participants in each group perceived verbal abuse and non-minor percentage perceived mobbing, but most of these events are not reported.

## Introduction

Violence is “the deliberate use of physical strength or power, either as a threat or an action against oneself, another person, a group or a community, which causes or is very likely to cause harm, death, psychological damage, growth disorders and deprivation^1^”. It is a public health issue which crosses every limit, regardless of race, age, socioeconomic status, education, creed or religion, sexual orientation or workplace, and is one of the top-ten causes of death and the fourth main cause of death in Chile^1^. It is a complex problem with biological, psychic, social and environmental roots^2^ which has reached epidemic levels, expanding towards several healthcare areas^1^, it is a complex and widespread phenomenon which affects the development of the communities, the quality of life and destroys the social tissue^3^. The broad range of moral codes in different countries makes violence one of the most delicate and hard-to-approach issues^4^. Workplace violence is “any unreasonable action, incident or behavior in which a person is attacked, threatened, humiliated or hurt by another person while performing his/her professional activities or as a consequence of such actions^5^”, workplace violence includes such events occurring while the person is going home from the workplace and vice versa which endanger the security, well-being or health of the worker are included^6^.

For many years, the healthcare area was free of violence episodes, just like education and social service areas^1^. However, this phenomenon has increased in the healthcare area^7^. Every day, healthcare staff members suffer violence events, and this phenomenon has become a big problem for those who work in healthcare^7^ ^-^ ^9^.

**Figure 1:**
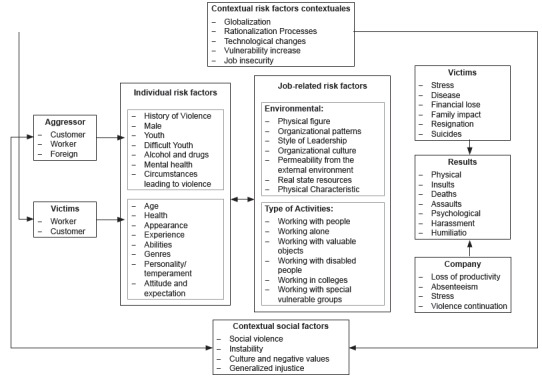
Interactive Model of Violence at Work. Chappell and Di Martino 2006, based on Poyner and Warne, 1988 p.7

An example of workplace violence is psychological violence, which is “the intentional use of power, including threats of physical force, against a person or group, which can result in physical, mental, spiritual, moral or social damage^1^ ^,^ ^3^ ^,^ ^10^”. It also includes verbal abuse as a an action that humiliates, disrespects and belittles the dignity and the value of a person and mobbing, which is a “repetitive type of violence in the workplace that is systematically used by people, regardless of gender, against other people and for a long time with the intention of causing damage; consequences can be devastating for the victims, who may suffer a number of psychological disorders^11^ ^-^ ^12^”. For many years, psychological violence has been underestimated; however, it occurs many times through repetitive attitudes that may be relatively unimportant, but in the long term, they can become a severe form of violence^5^ ^,^ ^10^ ^,^ ^13^. This research is based on the Interactive Model of Violence proposed by Chappell and Di Martino in 1998, which is based on the Model of Poyner and Warne; this model tries to explain workplace violence from a multifactorial point of view, where the interaction of several risk factors from the aggressor, the victim and the environment where these actions occur are related^2^ ^,^ ^14^. See Figure 1.

In Chile, the violence phenomenon has been studied from the perspective of users and healthcare providers within the hospital, where psychological violence has always been more common than physical violence in the workplace, this is similar to the information provided in other investigations in Latin America and the rest of the world^15^ ^-^ ^16^. On the other side, mobbing has been reported by healthcare providers within the hospital as an increasing phenomenon^12^ ^,^ ^17^ ^-^ ^18^.

Many international investigations have proven that most violence events occur in emergency services, psychiatric hospitals and nursing homes^19^. In Chile, pre-hospital care areas (SAMU, Emergency Medical Attention Service) have become a fundamental provider of emergency services in the country. The staff of SAMU ambulances consists of professionals (nurses, midwives, kinesiologists), paramedic technicians (nursing assistants), and directors, whose healthcare services are provided from the moment a potentially harmful health event is reported, wherever it is reported, until patient(s) is/are admitted into the emergency room. However, little is known about violence events that health care staff members suffer duringthe healthcare services provided outside the hospital; thereby, we intend to highlight workplace violence affecting paramedic technicians and professionals in order to prevent absenteeism by highly qualified staff which is not easy to replace during emergency situations occurring outside the hospital facility.

This study has the following objectives: to determine the perception of verbal abuse and mobbing and the associated factors of paramedic technicians and professionals of SAMU in three regions of the south of Chile. Research hypotheses are the following: H1: There are significant differences among regions regarding perception of verbal abuse and mobbing by professionals and paramedic technicians of SAMU during pre-hospital care. H2: there is a significant difference in perception of verbal abuse and mobbing among professionals and paramedic technicians of SAMU during pre-hospital care.

## Methods

Study design: A quantitative, descriptive and correlational study.

Analysis unit: Professional (nurse, midwive, kinesiologist) and paramedic technician (nursing assistant) working in pre-hospital care of chilean public healthcare service.

The study was performed on the entire population of professionals (n=74) y paramedic technician (n=360) in three regions of Chile.

Sample: For paramedic technician population a two staged stratified random sampling method was used. first, stratification was performed per region; then, communes where SAMU bases were located were randomly chosen, proportional affixation was applied accounting for a 5% error and a 95% confidence level, obtaining a sample size of n=148, which represents the three regions. 

Inclusion criteria: paramedic technician or professional who is willing to participate in the research, has signed an informed consent, and has been working in a SAMU unit for more than one year.

The sample studied was then composed of 72 professionals and 148 paramedic technician

Instrument: A questionnaire was applied “violence in the workplace in the health sector” proposed by WHO, ILO, CIE and PSI, which was validated in this investigation. For this, the original version of the questionnaire in the English language was translated into Spanish by a first translator. The translated Spanish version was subjected to a back-translation by a second driver different from the first. Finally, a third expert translator on the subject was responsible for combining and reviewing the original questionnaire with the latest version translated into English. The Spanish version was submitted to validation by experts for its comprehension and subsequent application in a pilot test. For construct validity, a principal component analysis was performed, which yields three factors that explain 86% of the variance. The application of the questionnaire during the pilot test to nurses and paramedical technicians (n = 52), allowed to improve the style of the questions applied to the prehospital care and the obtaining of the reliability of the questionnaire that was carried out through the analysis of internal consistency by calculation of the alpha of cronbach that gave a value of 0.91.

Data collection: the researcher personally visited the SAMU paramedic technicians and professionals from the three regions of Chile, during their working hours from October 2012 to May 2013. The self-administered questionnaire took 30 to 45 minutes to complete.

Analysis of data: The data analysis was performed in the statistical software SPSS v15.0. For the univariate analysis, descriptive statistics was applied and a chi-square test was used for inferential statistics.

Bias control: regarding theory and the questionnaire application, the study was thoroughly planned. The researcher applied the survey in the three regions.

Ethical considerations: For this study, we used the ethical principles proposed by Ezequiel Emmanuel, which are based on the CIOMS regulations; social value, scientific validity, favorable risk/benefit ratio, equitable selection of subjects, independent evaluation, informed consent and respect for the subjects participating in the investigation. The investigation was approved by the corresponding Ethics Committees of the healthcare institutions of each region involved in the study, as well as the Ethics Committee of the University of Concepción.

## Results

**Figure 2:**
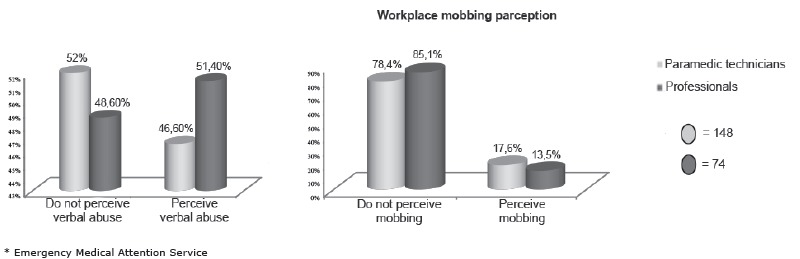
Verbal abuse and mobbing perception of professionals and paramedic technicians of SAMU*. Chile 2013

The biosociodemographic and employment characteristics and the history of violence, the professionals and the paramedic technicians are summarized in Table 1 and 2. The Verbal abuse and mobbing perception are summarized in Figure 2.

**Table 1:** Biosociodemographic and employment characteristics of professionals and paramedic technicians of SAMU*. Chile 2013

Biosociodemographic and employment characteristics	Professionals			Paramedic techinicians	
	Frequency (N=74)	%		Frequency (n=148)	%
Sex					
Female	33	44,6%		45	30%
Male	41	55,4%		102	69%
Age					
Average	33,6			36,6	
Standard Deviation	9,864			7,171	
Marital status					
Single	38	51,4%		64	43%
Married	22	29,7%		54	39%
Widow	0	0,0%		2	1%
Divorced/void	6	8,1%		13	9%
Years of work experience					
1 to 5 years	24	32,4%		48	32%
6 to 10 years	25	33,8%		37	25%
11 to 20 years	20	27,0%		36	24%
More than 20 years	5	6,8%		26	18%
Working in rotating shifts					
Yes	74	100,0%		145	98%
N°. of colleagues					
1 to 5	34	45,9%		73	49%
6 to 15	27	36,5%		56	38%
More than 15	13	17,6%		18	12%

* Emergency Medical Attention Service

**Tabela 2:** Description of variables associated with the violent incident of professionals and paramedic technicians of SAMU*. Chile 2013

Variables associated with the violent incident	Professionals			Paramedic techinicians	
	Frequency (n=74)	%		Frequency (n=148)	%
Concern for workplace violence					
Not concerned	12	16,2%		24	16%
Concerned	9	12,2%		27	18%
Very concerned	15	20,3%		53	36%
There are reporting procedures in the workplace					
Yes	36	48,6%		78	53%
Knowledge on the use of such procedures					
Yes	30	83,3%		57	73%
Personality traits					
Neurotic	2	2,7%		5	3,4%
Extroverted	7	9,5%		9	6,1%
Open to experiences	5	6,8%		7	4,7%
Kindness, pro-social	29	39,2%		42	28,4%
Responsibility	30	40,5%		83	56,1%
Active tobacco smoking					
Yes	29	39,2%		64	43,9%
Alcohol consumption					
Yes	43	61,4%		63	49,6%
Suffering child abuse					
Yes	10	13,50%		32	21,6%
Type of child abuse experienced					
Physical	2	20,0%		15	46,9%
Psychological	1	10,0%		6	18,8%
Physical and Psychological	3	30,0%		11	34,4%
Physical and sexual	2	20,0%		0	0,0%
All	2	20,0%		0	0,0%
Suffering from abuse in adulthood					
Yes	26	35,1%		39	26,4%
Type of abuse experienced during adulthood					
Physical	1	3,8%		5	12,8%
Psychological	21	80,8%		30	76,9%
Physical and Psychological	4	15,4%		4	10,3%

*Emergency Medical Attention Service

Regarding the frequency of verbal abuse in the workplace, both professionals (65.8%) and paramedic technicians (69.9%) who have suffered verbal violence state that this phenomenon only occurs sometimes, which is similar to the mobbing cases. The main verbal abusers identified are patients, relatives and general public (80%), while most mobbing perpetrators are staff members (30.8%) and external colleagues (emergency units that receive patients who are transferred by SAMU professionals and technicians) (34.6%). On the other side, professionals stated that the main verbal abusers are the immediate superior, members of the staff and external colleagues, in similar percentages (30%). Less than 3% of verbal abuse events (n=3) and 14% of mobbing events are investigated. I n only one case of mobbing, the aggressor was legally charged. Most affected people from both job categories state that reasons for not reporting verbal abuse and mobbing events include the following: because it was not important (32.2%) and because they thought it was useless (46%).

The statistically significant variables in relation to the variables under study are summarized in Table 3. No significant differences were found between the job categories and the studied regions.

**Table 3:** Factors associated to verbal abuse perception and mobbing in pre-hospital care (SAMU*)

Variables	Job category	Verbal Abuse	
Concern for workplace violence	Professionals (n=38)	11,157^†^	0,048^‡^
	Paramedic technicians (n=69)	15,220^†^	0,124^‡^
Adulthood abuse (More than 15 years)	Professionals (n=38)	2,865^†^	0,239^‡^
	Paramedic technicians (n=69)	15,644^†^	0,004^‡^
Intensity of psychological violence	Professionals (n=38)	15,032^†^	0,002^‡^
	Paramedic technicians (n=69)	33,094^†^	0,000^‡^
Variables	Categoría de trabajo	Mobbing	
Childhood abuse	Profesionales (n=10)	0,175^†^	0,676^‡^
	Técnicos paramédicos (n=26)	8,110^†^	0,004^‡^
Sex	Profesionales (n=10)	5,663^†^	0,017^‡^
	Técnicos paramédicos (n=26)	2,263^†^	0,132^‡^

*Emergency Medical Attention Service, † χ² Value, ‡ Significance level p

## Discussion

Pre-hospital care services in Chile were born during the 90’s with the goal of providing healthcare services outside the hospital facilities. Several international and local studies have stated that most workplace violence events occur in emergency rooms. The verbal abuse cases mentioned by both occupational categories are very similar to the cases reported in other studies^16^ ^,^ ^19^.Apparently, insults, teasing, and physical threats are common in Chile, South America and all over the world. This type of violence has the lowest-level legal sentences, contrary to cases of physical violence; because of this, people tend to accept insults without the abuser being legally penalized. Therefore, the abuser, knowing that he/she will not suffer any punishment, feels free to verbally abuse the victim^15^.

The main verbal abusers, in descending order, were: relatives of the customer, patients, public, a staff member, head or supervisor, and external colleagues. This ranking corresponds to both job categories, as mentioned in other studies performed in Argentina, Italy, Iran, Gambia, Jordania, and Chile^19^ ^-^ ^20^.

Public aggressiveness is one of the main variables identified in the job categories of pre-hospital care. It is important to remember that this healthcare service attends several places, some of them with high levels of aggressiveness in the population. This, together with the lack of knowledge about how healthcare system works, the waiting times for medical treatments, and the severity of the health problems, increases frustration among people receiving healthcare. However, for whatever reason, subjects of both job categories stated that it has become important to teach people about the functionality of pre-hospital care and how to make a proper use of this service, as well as shorten waiting times for patients in order to avoid exposing pre-hospital care professionals and technicians to insults by patients, relatives, and the general public^21^.

It is surprising to know that in both job categories, there is a low percentage of workers who report verbal violence events to their bosses; the reasons for not reporting can be summarized in “*it is useless*”and “*it was not important*”. These results are similar to the ones found in countries^22^.

In recent years, a new type of violence has been discovered; this type of violence increases systematically over time and its consequences can be devastating, it is called ‘mobbing’^12^ ^,^ ^23^ . Even though percentages are low in both job categories, it is important to note that this harassment dynamics occurs in pre-hospital care areas and affects fellow employees and their respective bosses. Both working categories state that this phenomenon occurs with low frequency in the workplace, but it causes stress to workers, as stated in the literature and by subjects in both job categories in this study.

It is important to note that sex variable was important for the perception of mobbing. Female workers are more vulnerable than male workers to suffer certain types of workplace violence^5^. The sex of the person in a male world, which characterizes this type of job, and the competitiveness they must face every day for a space in the workplace make them more likely to suffer this type of violence. 

On the other side, childhood abuse suffered by paramedic technicians was also related to the perception of mobbing. As mentioned above, childhood abuse is a variable that influences the way a person will react when he/she faces certain types of violence, as in this case^2^ ^,^ ^14^.

The fact that a low percentage of mobbing and verbal abuse victims decide to report these incidents is surprising. Generally, professionals and paramedic technicians do not report these violence events because “reporting is useless” and “because they think the event was not important”. It is important to notice that mobbing, despite being a continuous form of harassment, is considered by workers as unimportant even though it causes problems that may destroy labor relations^22^ ^-^ ^23^.

Harassment suffered by healthcare workers can be fatal for users, because it can affect workers’ performance, they may become emotionally vulnerable, and get tired after the constant attacks against them, resulting in poor healthcare services for users^2^ ^,^ ^5^ ^,^ ^13^ ^,^ ^24^.

When the workers from both job categories had the chance to ask for help regarding mobbing incidents, they stated that they had only been given chance to talk about the incident and that they had not received any professional help. This made professionals and paramedic technicians feel dissatisfied with the outcome of the events, and led other staff members of pre-hospital care to avoid reporting new mobbing episodes in their workplace, because they knew their workplace problems would not be solved, leading to a vicious circle in the work environment. In studies performed in other countries, one of the actions that are taken in order to stop these mobbing incidents and that seems worth for healthcare entities to take into account is the prevention of mobbing by encouraging workers to get along well with each other and to interact with each other in order to minimize these violence episodes, as well as by encouraging workers to report these violent incidents in the workplace.

## Conclusion

Pre-hospital care areas are not free of suffering violent incidents when providing healthcare services. Verbal abuse is the most prevalent, followed by mobbing events have appeared among workers of healthcare areas. It is absolutely necessary to encourage workers to report violence episodes that occur while providing healthcare services and those events that occur in the workplace in order to protect qualified professionals and paramedic technicians who attend medium to highly complex emergency calls, because the environment of these healthcare services must be free from any type of violence.
